# OpenAI's Sora and Google's Veo 2 in Action: A Narrative Review of Artificial Intelligence-driven Video Generation Models Transforming Healthcare

**DOI:** 10.7759/cureus.77593

**Published:** 2025-01-17

**Authors:** Mohamad-Hani Temsah, Rakan Nazer, Ibraheem Altamimi, Raniah Aldekhyyel, Amr Jamal, Mohammad Almansour, Fadi Aljamaan, Khalid Alhasan, Abdulkarim A Temsah, Ayman Al-Eyadhy, Bandar N Aljafen, Khalid H Malki

**Affiliations:** 1 Pediatrics, College of Medicine, King Saud University, Riyadh, SAU; 2 Pediatric Intensive Care Unit, King Saud University Medical City, Riyadh, SAU; 3 Cardiac Surgery, King Khalid University Hospital, King Saud University, Riyadh, SAU; 4 Medicine, College of Medicine, King Saud University, Riyadh, SAU; 5 Medical Education and Simulation, College of Medicine, King Saud University, Riyadh, SAU; 6 Family and Community Medicine, College of Medicine, King Saud University, Riyadh, SAU; 7 Critical Care, College of Medicine, King Saud University, Riyadh, SAU; 8 Pediatric Nephrology, College of Medicine, King Saud University, Riyadh, SAU; 9 Dentistry, Specialized Medical Center Hospitals, Riyadh, SAU; 10 Otolaryngology-Head and Neck Surgery, College of Medicine, King Saud University, Riyadh, SAU

**Keywords:** deepfake, ethical concerns in ai, generative ai, google veo, llm patient education videos, medical education technology, openai sora, patient education videos, telemedicine ai, text-to-video ai

## Abstract

The rapid evolution of generative artificial intelligence (AI) has introduced transformative technologies across various domains, with text-to-video (T2V) generation models emerging as transformative innovations in the field. This narrative review explores the potential of T2V AI generation models used in healthcare, focusing on their applications, challenges, and future directions. Advanced T2V platforms, such as Sora Turbo (OpenAI, Inc., San Francisco, California, United States) and Veo 2 (Google LLC, Mountain View, California, United States), both announced in December 2024, offer the capability to generate high-fidelity video contents. Such models could revolutionize healthcare by providing tailored videos for patient education, enhancing medical training, and possibly optimizing telemedicine. We conducted a comprehensive narrative literature search of databases including PubMed and Google Scholar, and identified 41 relevant studies published between 2020 and 2024. Our findings reveal significant possible benefits in improving patient education, standardizing customized medical training, and enhancing remote medical consultations. However, critical challenges persist, including risks of misinformation (or deepfake), privacy breaches, ethical concerns, and limitations in video authenticity. Detection mechanisms for deepfakes and regulatory frameworks remain underdeveloped, necessitating further interdisciplinary research and vigilant policy development. Future advancements in T2V AI generation models could enable real-time healthcare visualizations and augmented reality training. However, achieving these benefits will require addressing accessibility challenges to ensure equitable implementation and prevent potential disparities. By addressing these challenges and fostering collaboration among stakeholders, healthcare systems and AI technologists, T2V AI generation models could transform global healthcare into a more effective, universal, and innovative system while safeguarding against its potential misuse.

## Introduction and background

The introduction of advanced artificial intelligence (AI) tools has revolutionized multiple domains, with text-to-video (T2V) generation models emerging as a transformative innovation in the field. Cutting-edge T2V platforms, like the widely-available Sora Turbo (OpenAI, Inc., San Francisco, California, United States) and Veo 2 (Google LLC, Mountain View, California, United States) that were revealed in December 2024, offer more abilities to generate high-quality video contents that are generated by text input [1,2]. Platforms such as Sora and other models have demonstrated the potential to create high-fidelity video content from textual inputs, leading to significant applications in education, healthcare, and beyond [3,4]. T2V AI could be particularly transformative in healthcare, where it may be employed to enhance patient education, medical training, and procedural simulation [4,5]. This innovation builds upon previous AI capabilities, such as image and speech generation, integrating visual and auditory modalities into cohesive and instructive video outputs [5].

Healthcare, characterized by complex information delivery and procedural instruction, stands to benefit immensely from T2V AI models. AI-generated videos can simplify patient communication, enhance physician training, and potentially reduce costs by automating the creation of high-quality educational content [4,6]. These tools have already shown promise in creating patient-specific instructional videos, aiding remote consultations, and even simulating complex medical procedures [7-9]. However, while the potential is vast, challenges still remain. Ethical concerns regarding misinformation, bias, and the accuracy of AI-generated medical content are critical considerations [10,11]. Additionally, technical limitations such as inaccuracies in medical imaging or the inability to fully replicate human behaviors highlight the necessity for further refinement and oversight [12-14].

Advanced T2V AI generation models may offer an unprecedented opportunity to transform healthcare by simplifying complex information delivery, enhancing educational tools, and improving patient-specific communication. This comprehensive narrative review synthesizes recent advancements to evaluate the potential benefits and challenges, ensuring these tools are responsibly integrated into clinical practice [15,16].

This extensive narrative review explores the application of T2V generation models in healthcare by exploring publications from PubMed and Google Scholar between 2020 and 2024. This review highlights the potential benefits, evaluates the risks, and offers a framework for integrating these technologies responsibly into healthcare practice. It aims to provide a comprehensive understanding of advancements in T2V AI models by exploring their applications, challenges, and relevance to key areas such as medical education, patient engagement, public health campaigns, and ethical or privacy considerations.

## Review

Methods

Similar to the method used in our previous review on early ChatGPT [17], an extensive literature search was conducted by two researchers (MHT and KAH) on December 31, 2024, to explore the role of AI-generated videos in healthcare. The primary database for the search was PubMed, with a focus on articles published in English from Jan 2020 to December 2024. Search terms included “AI-generated videos", “AI-generated fake videos", “Sora video", “Veo AI video", “Veo Google", “Veo2 AI", “artificial intelligence text-to-video", “AI text-to-video medical education", “AI-generated video simulation", “deep fake video", “AI-generated video ethics", and “Privacy AI-generated videos".

The inclusion criteria were studies published between 2020 and 2024, written in English, and directly addressing healthcare applications of AI-generated videos, including medical education, patient engagement, public health campaigns, and ethical or privacy considerations. The PubMed search initially yielded 66 articles across the various keywords. After screening titles and abstracts, 39 unique papers were included in the review. Duplicate studies and papers lacking relevance to healthcare contexts were excluded. For example, 23 papers were initially identified under "AI-generated videos," but only 18 were deemed relevant after screening. Similarly, 12 papers were found using the keyword "deep fake video," with 10 included for their focus on ethical, legal, and practical implications. The search for "Sora video" resulted in 12 articles, of which eight were included due to their exploration of Sora’s applications in medical simulation and patient education. Keywords related to "Veo", including "Veo AI video", "Veo Google", and "Veo2 AI" returned no results on PubMed.

To address the lack of publications on Veo, supplemental searches were conducted on Google Scholar using similar keywords. This yielded two preprints focusing on deepfake detection, both of which were included to provide additional perspectives on emerging technologies and ethical considerations [11,18].

Results

In total, 41 papers were included in this comprehensive narrative review: 39 peer-reviewed studies from PubMed and two preprints sourced from Google Scholar. These studies encompassed a range of applications, risks, and technological advancements in T2V AI generation models, with findings categorized into five key areas: patient education, medical training, telemedicine, ethical considerations, and technology limitations. The comprehensive nature of this review offers a nuanced understanding of the current state and future potential of T2V AI generation models in healthcare, as illustrated in Table 1.

**Table 1:** Key findings and examples of T2V AI applications in healthcare AI: artificial intelligence; LASIK: laser-assisted in situ keratomileusis; PRK: photorefractive keratectomy, T2V: text-to-videos

Category	Key Findings and Examples	Authors	Year	Reference
Patient Education	Tailored videos enhance patient understanding of procedures like LASIK and PRK.	Zhang et al.	2024	[4]
		Macri et al.	2024	[19]
Medical Training	Simulations of neurosurgical procedures standardize training; FlexLip improves video authenticity.	Oneață et al.	2022	[12]
		McLean and Pineda	2024	[20]
Telemedicine	AI-generated videos streamline consultations and bridge gaps in underserved areas.	Waisberg et al.	2024	[3]
		Chen et al.	2024	[11]
Ethical Considerations	Risks include misinformation, deepfake, privacy breaches, and biases in generated content.	Li et al.		[8]
		Shahzad et al.	2022	[10]
		Chen et al.	2024	[11]
Technology Limitations	Challenges include inaccuracies in medical imagery and insufficient de-identification methods, high energy demands.	Oneață et al.	2022	[12]
		Javan et al.	2024	[13]

Discussion

**Figure 1:**
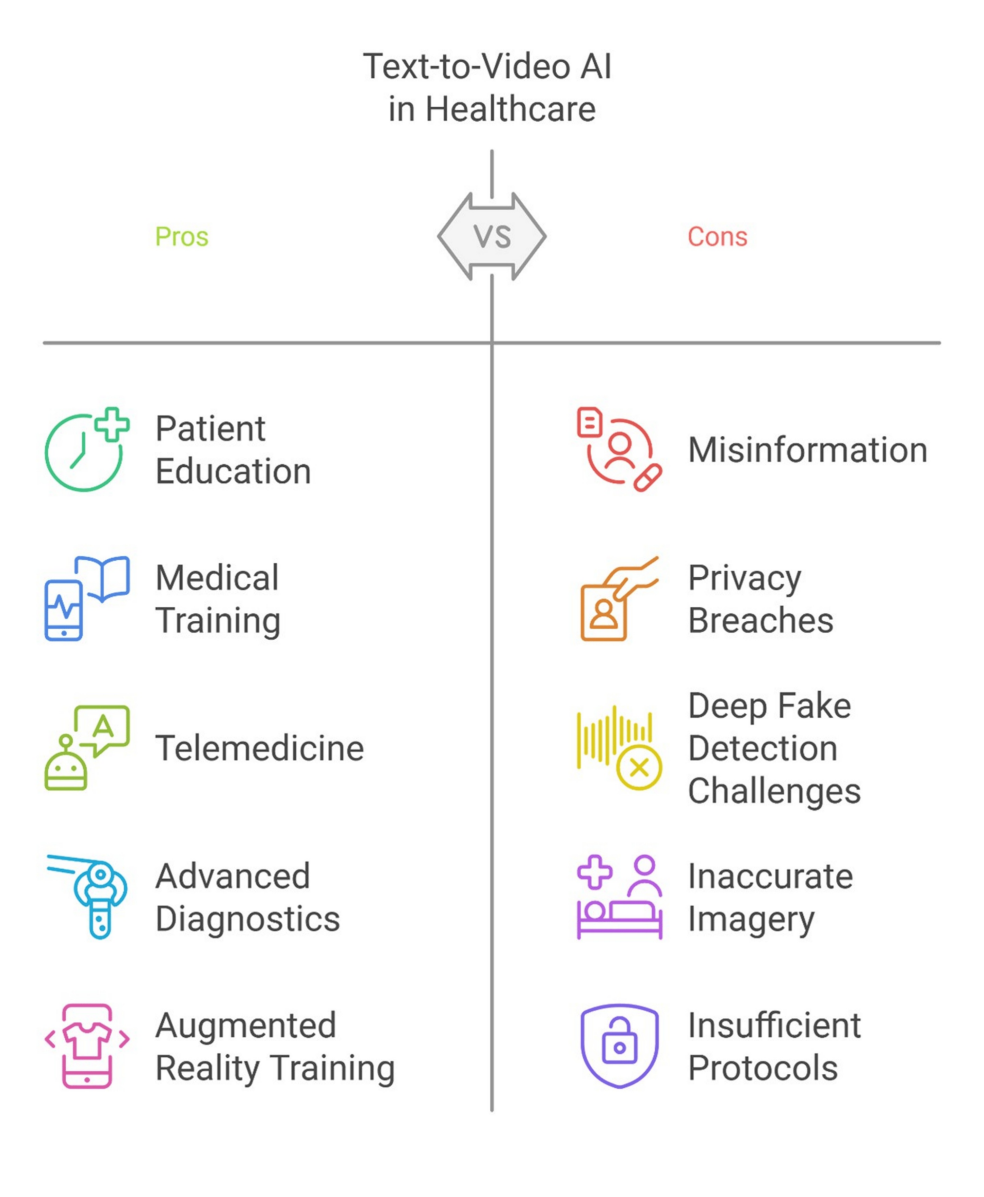
The pros and cons of text-to-videos generated by artificial intelligence AI: artificial intelligence; T2V: text-to-video Image Credit: Mohamad-Hani Temsah, Author

By integrating peer-reviewed studies and gray literature, this review provides a balanced analysis of the field’s current state and potential future directions, with several pros and cons (Figure 1).

Applications in patient education

One of the most immediate benefits of T2V AI generation models is its potential to enhance patient education. By generating tailored video content, patients can receive visually engaging, easy-to-understand instructions about their medical conditions and treatments. AI-generated videos for surgical procedures such as laser-assisted in situ keratomileusis (LASIK) and photorefractive keratectomy (PRK) have demonstrated success in increasing patient knowledge while maintaining high standards of script accuracy and visual clarity [5,19]. The use of platforms like Sora has further facilitated the creation of realistic and interactive videos, particularly in ophthalmology and corneal refractive surgery education [5]. Additionally, systems like FlexLip, a controllable text-to-lip system, enhance accessibility by ensuring precise lip synchronization for more relatable and trustworthy content [12].

Revolutionizing medical education and training

T2V AI generation models have the potential to redefine medical education by creating immersive learning environments. Platforms like Sora have demonstrated their capacity to simulate complex neurosurgical procedures, offering trainees standardized yet adaptable scenarios for practice [20]. In surgical education, AI-generated videos are increasingly employed in training systems like those focused on laparoscopic procedures, where AI has successfully matched or exceeded human performance in skill assessments [21]. FlexLip’s advancements in synchronizing speech with realistic lip movements further enhance the authenticity of these educational tools [12]. Another study on AI and gamification in laparoscopic cholecystectomy simulation (LapBot Safe Chole) showed the effective integration of AI and videos into a fun educational game application [22]. By integrating AI-generated videos into curricula, medical institutions can improve diagnostic accuracy and procedural competency, equipping trainees with essential skills in a cost-effective manner [23].

Enhancing telemedicine and remote care

In the realm of telemedicine, T2V AI generation models could provide solutions for enhancing patient-provider communication. Platforms like “Tele-Untethered” (University of California San Diego, Sandiego, California, United States) integrate AI-generated video content into telemedicine workflows, facilitating remote consultations and personalized follow-up care [24]. The inclusion of AI-generated videos in telehealth consultations ensures consistent and visually engaging communication, making complex medical advice more comprehensible to patients [3]. This capability is particularly important in underserved areas where access to healthcare professionals is limited. By enabling virtual demonstrations of medical procedures or health monitoring techniques, T2V AI generation models could bridge gaps in healthcare delivery, ensuring equitable access to quality care [11].

Ensuring equitable access to AI-driven T2V technologies in healthcare

Though integrating T2V AI models into healthcare may provide opportunities to enhance patient education and streamline telemedicine, ensuring equitable access to these technologies remains crucial to prevent exacerbating existing healthcare disparities [25,26]. Challenges such as limited infrastructure, high costs, energy consumption, and lack of technical expertise in underserved regions can impede the widespread adoption of AI-driven tools. Addressing these barriers requires targeted investments, policy initiatives, and collaborative efforts among stakeholders to promote fairness in universal and sustainable AI practices in healthcare delivery [27].

Addressing ethical and privacy concerns

While the benefits are significant, T2V AI generation models also present ethical challenges. Misinformation, or deepfakes, remains a critical risk, as AI-generated audio or video could propagate inaccuracies or misleading content [10,28-30]. Additionally, privacy concerns arise from the potential misuse of patient-specific data in generating educational or clinical content. Studies examining de-identification protocols, such as the use of face-swapping technologies for privacy preservation, highlight the importance of robust safeguards to protect sensitive information [8,11]. Sora’s application in critical care medicine further emphasizes the need for secure handling of data to ensure patient confidentiality [4].

Challenges in deepfake detection and verification

The authenticity of AI-generated content is a growing concern, particularly with the rise of deepfake technologies [31,32]. Detection methods, such as DeCoF [18] or DeMamba artifact analysis [11] and advanced signal processing techniques, may offer promising avenues for verifying video integrity but require broader adoption and refinement. Deepfake identification systems, like those using residual networks and frequency analysis, have been proposed as essential tools in preventing the misuse of T2V technology in healthcare settings [33]. These efforts underscore the importance of interdisciplinary and continued collaboration among AI technologists, clinicians, and policymakers to establish standards and ensure the reliability of novel deepfake detection techniques [34-42].

Self-correction and content check mechanisms

To maximize the reliability of T2V AI generation models, incorporating mechanisms for self-correction and content verification is essential. Future systems should feature built-in feedback loops that allow continuous improvement of output quality. For example, real-time discrepancy detection between generated content and verified medical databases could flag potential inaccuracies before dissemination [43]. Vigilant and continuous evaluation and monitoring are critical for ensuring system reliability and optimizing outcomes, particularly in complex healthcare contexts. Additionally, integration with peer-review systems and domain-specific AI modules should help verify medical terminology and ensure visual accuracy, reducing the risk of misinformation [44].

Future potentials in advanced healthcare applications

Looking ahead, the integration of T2V AI generation models into advanced healthcare applications is poised to expand. Systems like Sora are exploring possibilities such as real-time three-dimensional visualizations and interactive training modules that incorporate augmented reality [3]. In intensive care medicine, Sora has shown the potential to improve the visualization of physiological processes and simulate critical scenarios for training purposes [4]. Additionally, the ability to simulate rare clinical scenarios offers trainees exposure to diverse cases, improving preparedness for real-world challenges [20,23]. The potential to integrate these tools into patient monitoring and diagnostics further expands their applicability in modern medicine.

Pros and cons of T2V in healthcare

The studies reviewed in this narrative highlight several potential benefits and challenges associated with T2V AI generation models in healthcare. On the positive side, these technologies demonstrate potential for enhancing patient education through tailored, visually engaging videos, improving medical training with realistic simulations, and facilitating telemedicine by streamlining remote consultations. However, critical challenges persist, including risks of misinformation through deepfake content, privacy concerns related to data usage, and ethical dilemmas surrounding the authenticity of AI-generated videos. Additionally, technical limitations, such as inaccuracies in medical imaging and accessibility barriers in underserved regions, highlight the need for further refinement and equitable implementation of these technologies. Addressing these pros and cons is essential for maximizing the benefits of T2V AI models while minimizing potential risks.

Limitations and opportunities for further research

Despite the promise of T2V AI generation models, its adoption is hindered by technical, ethical, and logistical challenges. Current models often struggle with inaccuracies and “hallucinations” in replicating medical imagery or human behavior, limiting their applicability in high-stakes scenarios [12-14,45,46]. Additionally, these models are prone to biases arising from training data that may not adequately represent diverse patient populations, potentially leading to outputs that lack inclusivity or accuracy in addressing varied medical needs [47,48]. Moreover, regulatory frameworks for AI-generated content in healthcare are still evolving, necessitating comprehensive guidelines to ensure responsible use [3,49,50]. Also, as many current T2Z AI models generate videos without audio, the addition of AI-generated audio needs further research to ensure their reliability [29]. Longitudinal studies are needed to evaluate the long-term efficacy and safety of T2V AI generation models, particularly in patient care and medical education.

## Conclusions

This review highlights that T2V AI generation models are a promising early-stage innovation in healthcare, with significant potential to enhance medical education and patient engagement. However, challenges such as misinformation, deepfakes, privacy concerns, and ethical issues must be addressed through robust safeguards and equitable implementation. A balanced collaboration among policymakers, AI developers, and healthcare professionals is essential to establish ethical standards and ensure the responsible adoption of these transformative technologies.
